# WGS-Based Prediction and Analysis of Antimicrobial Resistance in *Campylobacter jejuni* Isolates From Israel

**DOI:** 10.3389/fcimb.2020.00365

**Published:** 2020-08-13

**Authors:** Assaf Rokney, Lea Valinsky, Katleen Vranckx, Noa Feldman, Vered Agmon, Jacob Moran-Gilad, Miriam Weinberger

**Affiliations:** ^1^Central Government Laboratories, Israel Ministry of Health, Jerusalem, Israel; ^2^Applied Maths NV, Sint-Martens-Latem, Belgium; ^3^Department of Health Systems Management, School of Public Health, Faculty of Health Sciences, Ben Gurion University of the Negev, Be'er Sheva, Israel; ^4^Infectious Diseases Unit, Shamir (Assaf Harofeh) Medical Center, Zerifin, Israel; ^5^Sackler Faculty of Medicine, Tel Aviv University, Tel Aviv, Israel

**Keywords:** *Campylobacter jejuni*, antimicrobial resistance, antimicrobial-susceptibility testing, bioinformatics, whole-genome sequencing

## Abstract

Rapid developments in the field of whole genome sequencing (WGS) make *in silico* antimicrobial resistance (AMR) a target within reach. *Campylobacter jejuni* is a leading cause of foodborne infections in Israel with increasing rates of resistance. We applied WGS analysis to study the prevalence and genetic basis of AMR in 263 *C. jejuni* human and veterinary representative isolates retrieved from a national collection during 2003–2012. We evaluated the prediction of phenotypic AMR from genomic data. Genomes were screened by the NCBI AMRFinderPlus and the BioNumerics tools for acquired AMR genes and point mutations. The results were compared to phenotypic resistance determined by broth microdilution. The most prevalent resistant determinants were the multi-drug efflux transporter gene *cmeABC* (100%), the tetracycline resistance *tet(O)* gene (82.1%), the quinolone resistance g*yrA* T861 point mutation (75.7%), and the *aadE* streptomycin resistance gene. A variety of 12 known β lactam resistance genes (*bla*_OXA_ variants) were detected in 241 (92%) isolates, the most prevalent being *bla*_OXA−193_, *bla*_OXA−461_, and *bla*_OXA−580_ (56, 16, and 7%, respectively). Other aminoglycoside resistance genes and the macrolide resistance point mutation were rare (<1%). The overall correlation rate between WGS-based genotypic prediction and phenotypic resistance was 98.8%, sensitivity, specificity, positive, and negative predictive values being 98.0, 99.3, 99.1, and 98.5%, respectively. wgMLST-based phylogeny indicated a high level of clonality and clustering among the studied isolates. Closely related isolates that were part of a genetic cluster (single linkage distance ≤ 15 alleles) based on wgMLST phylogeny mostly shared a homogenous AMR determinant profile. This was observed in 18 of 20 (90.0%) clusters within clonal complex-21, suggesting clonal expansion of resistant isolates. Strong association to lineage was noted for the *aadE* gene and the various *bla*_OXA_ genes. High resistance rates to tetracycline and quinolones and a low resistance rate to macrolides were detected among the Israeli *C. jejuni* isolates. While a high genotypic-phenotypic correlation was found, some resistance phenotypes could not be predicted by the presence of AMR determinants, and particularly not the level of resistance. WGS-based prediction of antimicrobial resistance in *C. jejuni* requires further optimization in order to integrate this approach in the routine workflow of public health laboratories for foodborne surveillance.

## Introduction

Whole genome sequencing (WGS) has revolutionized foodborne pathogen surveillance practices (Jagadeesan et al., 2019). This has become achievable due to the rapid developments in high-throughput sequencing technologies, allowing affordable, real-time, large-scale WGS of foodborne pathogens. This technology can produce a large amount of fast, highly accurate, and reliable information permitting species identification and typing, phylogenetic analyses, outbreak investigation, and determination of virulence and resistance traits as a one-stop-shop (Motro and Moran-Gilad, 2017; Ribot et al., 2019). WGS is increasingly introduced as the primary method for foodborne pathogen surveillance in public health laboratories, replacing the preexisting conventional methods for species identification and typing such as serotyping, pulsed-field gel electrophoresis (PFGE), multilocus variable-number tandem-repeat analysis (MLVA), or multilocus sequence typing (MLST) (Inns et al., 2017; Nadon et al., 2017; Revez et al., 2017; Ribot et al., 2019). The superior discriminatory power of WGS compared to the above typing methods has been established in the detection, investigation, and source tracking of local, multi-state, and multi-national foodborne outbreaks (Crowe et al., 2017; Inns et al., 2017; Llarena et al., 2017; Rumore et al., 2018; Gerner-Smidt et al., 2019; Kubota et al., 2019) and is becoming the new gold standard for molecular surveillance of foodborne pathogens (Moran-Gilad, 2017; Gerner-Smidt et al., 2019).

*Campylobacter jejuni* is a major foodborne pathogen and a leading cause of human gastroenteritis globally, thus it is the target for surveillance in many countries. WGS is rapidly becoming the first line method for *Campylobacter* subtyping in many public health laboratories, mostly based on whole genome (wg) multilocus sequence typing (MLST) (wgMLST) and core genome MLST (cgMLST) (Nadon et al., 2017; Ribot et al., 2019).

The incidence of *C. jejuni* infections in Israel is among the highest in industrialized countries, with an alarming burden on young children (Weinberger et al., 2013). wgMLST-based epidemiologic analyses of Israeli *C. jejuni* isolates suggest that poultry and cattle are likely food sources of infection in Israel, with different strains persisting over years throughout the food chain (Rokney et al., 2018). The analyses also portrayed the spectrum of virulence determinants harbored by the Israeli isolates.

*Campylobacter* species are becoming increasingly resistant to the antibiotics relevant to the treatment of clinical infection. Resistance to fluoroquinolones is widespread in both *C. jejuni* and *C. coli*. In the European Union (EU) countries the overall resistance rate to ciprofloxacin among *C. jejuni* human isolates was 58% in 2017, with the highest rates reported from Portugal (97%), Lithuania (92%), Spain (87%), Estonia (84%), and Cyprus (80.0%). Resistance among *C. coli* human isolates was even higher, reaching an overall rate of 63% and highest rates in Estonia (100%), Portugal (100%), Lithuania (97%), and Spain (96%) (European Food Safety Authority and European Centre for Disease Prevention and Control, 2019). Close to 90% resistance rates were also noted in Peru and China (CDC, 2018; Sproston et al., 2018). Resistance to macrolides is also increasing globally, although not at the same pace. It remains relatively low for *C. jejuni* in Europe (<3% overall) and the United States (<5%), but higher for *C. coli* (<15% in the United States and ~13% in EU countries) (CDC, 2018, 2019; Florez-Cuadrado et al., 2018; European Food Safety Authority and European Centre for Disease Prevention and Control, 2019). Resistance among isolates from farm and wild animals may be even higher (CDC, 2018, 2019; Florez-Cuadrado et al., 2018; European Food Safety Authority and European Centre for Disease Prevention and Control, 2019). *Campylobacter* is listed in the most recent report from the Centers for Disease Control and Prevention (CDC) Antibiotic Resistance Threats in the United States (CDC, 2019) and named by the World Health Organization (WHO) as one of the 12 bacteria that pose the greatest threat to human health due to their antibiotic resistance (Sproston et al., 2018).

In Israel, the reported data on *Campylobacter* resistance is scarce. In a study from Northern Israel, a sample of 66 stool isolates from the years 2015–2016 was tested by both the Sensititre® broth microdilution plate and the E-test methods. Resistance rates to fluoroquinolones were alarmingly high for both *C. jejuni* and *C. coli*, reaching >95%, but were low for macrolides (<3%) (Azrad et al., 2018). Similar results were reported in the most recent periodic publication of the Government Central Laboratories, Israeli Ministry of Health, Jerusalem (available at https://www.health.gov.il/PublicationsFiles/LAB_JER2017.pdf, last accessed on February 29, 2020).

The impressive developments in fast and affordable bacterial WGS, the availability of appropriate bioinformatics tools and comprehensive databases, make *in silico* antimicrobial resistance (AMR) detection a target within reach (Hendriksen et al., 2019). While many hindrances still exist (Ellington et al., 2017), the use of WGS to detect AMR genes and infer phenotypes has shown excellent results in some recent studies (Feldgarden et al., 2019; Hendriksen et al., 2019; Su et al., 2019), including *Campylobacter* (Zhao et al., 2016; Whitehouse et al., 2018; Painset et al., 2020).

In this study, WGS analysis of representative *C. jejuni* Israeli isolates retrieved from a national collection was applied to study the genetic basis and dynamics of genotypic AMR and to evaluate the role of WGS in predicting phenotypic AMR.

## Materials and Methods

### Study Isolates

We studied 263 *C. jejuni* strains representing the major lineages in Israel as identified by MLST and collected over a 10-year period (2003–2012), as previously described (Weinberger et al., 2016; Rokney et al., 2018). The majority of isolates were recovered from human clinical specimens (*n* = 239), and the remaining were recovered from poultry and bovine sources (Supplementary Table 1).

### Antimicrobial Susceptibility Testing

A sample of 219 strains was tested for antimicrobial susceptibility to erythromycin, ciprofloxacin, tetracycline, gentamicin, nalidixic acid, and streptomycin (1,314 tests). The analysis was performed by broth microdilution at the National Reference Center for *Campylobacter*. Isolates were cultured on tryptic soy agar plates with 5% defibrinated sheep blood (Hylabs, Israel) and incubated at 42°C under microaerobic conditions (5% O_2_, 10% CO_2_) for 16–24 h. Broth microdilution was performed using the EUCAMP2 plate (Sensititre^TM^, TREK diagnostic systems LTD.), according to manufacturer's instructions. Minimum inhibitory concentrations (MIC) were interpreted using the European Committee on Antimicrobial Susceptibility Testing (EUCAST 2019) breakpoint for erythromycin (*R* > 4 μg/mL), ciprofloxacin (*R* > 0.5 μg/mL), and tetracycline (*R* > 2 μg/mL). The National Antimicrobial Resistance Monitoring System for Enteric Bacteria (NARMS, 2018) breakpoints were applied for gentamicin (*R* ≥ 4 μg/mL) and for nalidixic acid (≥32 μg/ μg/mL). Streptomycin resistance was determined according to the EUCAST epidemiological cut-off values (ECOFFs) (*R* > 4 μg/mL). Multidrug resistance was defined as non-susceptibility to at least one agent in three or more antimicrobial classes. Broth microdilution results are detailed in Supplementary Table 2.

### WGS Analysis

DNA extraction was performed on the automated QIAsymphony SP platform (Qiagen, Hilden, Germany) and quantified by the Qubit 2.0 Fluorometer (Life Technologies, Carlsbad, CA, USA). DNA libraries were prepared from 1 ng of purified DNA using Nextera XT DNA Sample Prep Kit and Nextera XT Index Kit (Illumina, Inc., San Diego, CA, USA). Libraries were subject to short read sequencing PE 250bpx2, aiming at >100X coverage. The reads were *de novo* assembled by SPAdes 3.7.1 on the BioNumerics calculation engine (Applied Maths, Belgium). Whole-genome multi locus sequence typing (wgMLST) analysis of assembly-free and assembly-based allele calls for 3,529 loci according to the BioNumerics *Campylobacter* scheme was performed on the BioNumerics calculation engine (Applied Maths, Belgium). Assemblies are available at the *Campylobacter* PubMLST database with IDs 76153-76415.

Phylogeny was inferred by calculating a minimal spanning tree based on wgMLST or MLST allelic profiles. Identification of potential clusters was based on a cutoff of ≤ 15 wgMLST allele difference (Rokney et al., 2018; Schürch et al., 2018).

Assemblies were screened by the NCBI AMRFinderPlus tool (version 3.1.1b) for known acquired AMR genes and resistance-conferring point mutations in *Campylobacter* (available at https://ftp.ncbi.nlm.nih.gov/pathogen/Antimicrobial_resistance/AMRFinder/, last accessed on February 29, 2020) (Feldgarden et al., 2019). The BioNumerics version 7.6.3 gene extraction tool (Applied Maths, Belgium) was used to screen assemblies for the gene cluster that includes *cmeR* and the multidrug efflux pump *cmeABC* (Accession AF466820). The G → T transversion in the promoter upstream of *bla*_OXA−193_ was screened with accession NG049489.1 as reference.

### Genotypic–Phenotypic Comparisons

The WGS-predicted resistance was compared with the phenotypic resistance for six clinically relevant antimicrobial agents (erythromycin, ciprofloxacin, nalidixic acid, tetracycline, gentamicin, streptomycin). The following calculations were performed: correlation (calculated as the sum of true positive and true negative divided by all tested isolates), sensitivity (calculated by dividing true positive by the sum of true positive and false positive), specificity (calculated by dividing true negative by the sum of true negative and false negative), positive predictive value (PPV) (calculated by dividing true positive by the sum of true positive and false negative), and negative predictive value (NPV) (calculated by dividing true negative by the sum of true negative and false positive).

### Statistical Methods

Poisson regression models accounting for overdispersion were used to study the annual trends of the incidence rate for *Campylobacter* AMR determinants. In these models, the yearly counts of isolates positive for the AMR determinants were the dependent variable; the calendar year was the independent variable, and the total number of tested isolates was the offset parameter.

### Ethical Consideration

The study was approved by the local Ethical Committee at Shamir (Assaf Harofeh) Medical Center, Zerifin (#160/11), Israel and the local Ethical Committee of Tel Aviv University, Tel Aviv, Israel.

## Results

### WGS of Study Isolates

**Figure 1:**
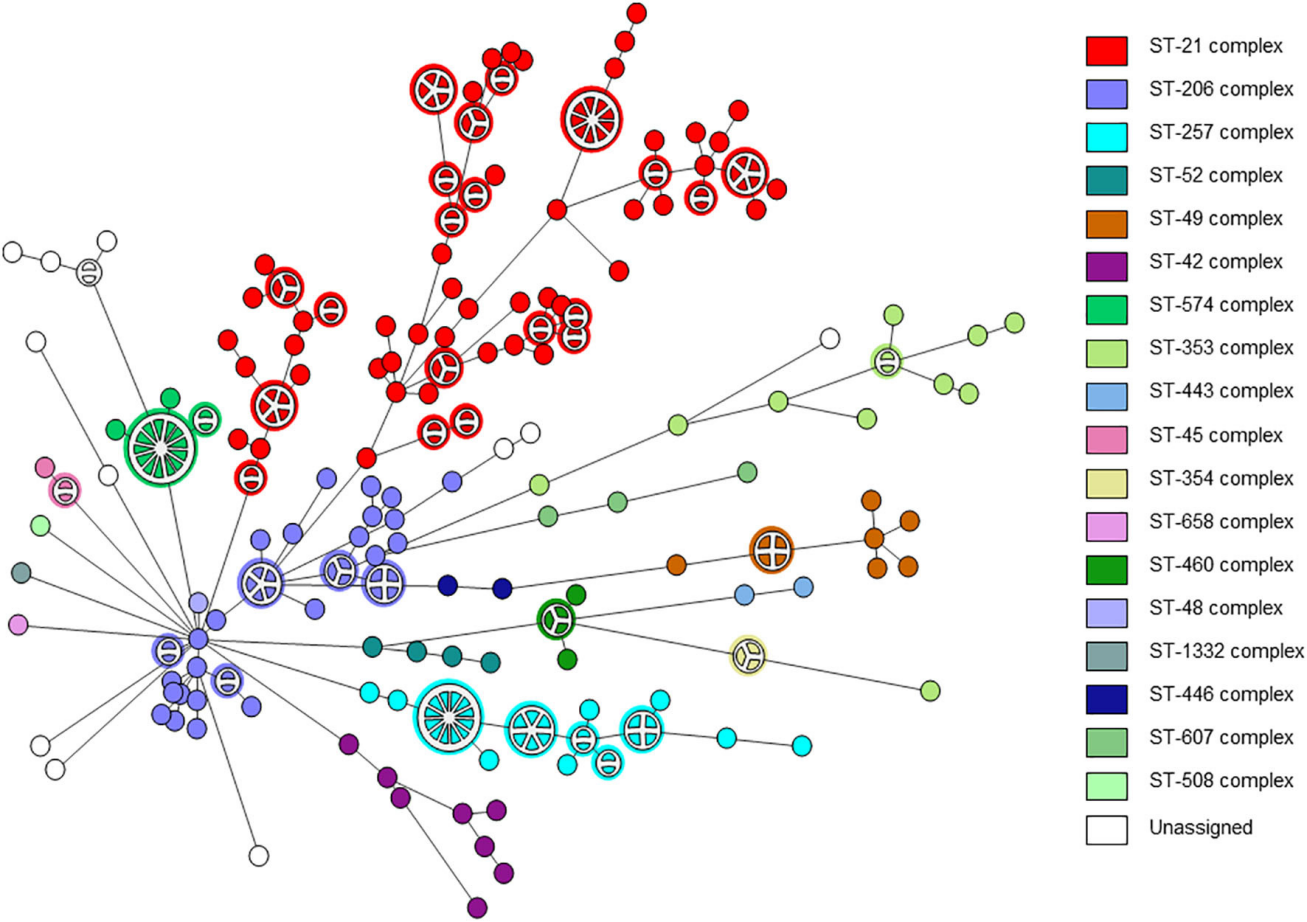
wgMLST-based phylogeny of 263 *C. jejuni* isolates. The minimum spanning tree is based on wgMLST analyses of 239 clinical and 24 veterinary isolates. Isolates are represented by circles connected by branches proportional to the allelic distance. The distribution of clonal complexes among the studied population is denoted by color. Partitioned nodes represent closely clustered isolates (≤ 15 allelic distance threshold).

A total of 263 *C. jejuni* isolates were analyzed by whole genome sequencing (Supplementary Table 1). The strains were collected from human (*n* = 239) and veterinary (*n* = 24) sources over a decade. The strains represented the *C. jejuni* population in Israel and included 18 clonal complexes (CCs) and 51 sequence types (STs), including the major lineages in Israel (Figure 1). Isolate sources, lineages, and the quality metrics of reads and assemblies are detailed in Supplementary Table 1.

### Genomic Prediction of AMR

AMRFinderPlus analysis detected a total of 21 genetic AMR determinants among the 263 *C. jejuni* studied sequences. These included 18 acquired AMR genes and 3 resistance-conferring point mutations (Table 1). The *tet(O)* gene, predicting resistance to tetracycline (Feldgarden et al., 2019), was the most prevalent AMR gene occurring in 82% of the isolates. The gene *aadE (ant(6)-Ia)* predicting resistance to streptomycin (Feldgarden et al., 2019) was detected in 19% of the isolates, while the genes *aph(3*′*)-IIIa* (V01547.1) predicting resistance to kanamycin and amikacin (Feldgarden et al., 2019), *aad9* (AY701528.1) predicting resistance to spectinomycin, and *sat4* (AJ489618.1) predicting resistance to streptothricin (Shen et al., 2017) were only rarely found (0.8, 0.4, and 0.8%, respectively).

**Table 1:** Antimicrobial resistance genetic determinants detected by AMRFinderPlus tool in 263 *C. jejuni* strains.

**Antimicrobial class**	**Genetic AMR determinant (gene symbol)**	**Description**	**No. of positive isolates (%)**
Tetracyclines	*tet(O)*	Acquired AMR gene	216 (82.1)
Quinolones	*gyrA* T86I	Point mutation	199 (75.7)
β-Lactams	*bla*_OXA−193_	Acquired AMR gene	147 (55.9)
	*bla*_OXA−461_	Acquired AMR gene	41 (15.6)
	*bla*_OXA−580_	Acquired AMR gene	18 (6.8)
	*bla*_OXA_^a^	Acquired AMR gene	16 (6.1)
	*bla*_OXA−184_	Acquired AMR gene	4 (1.5)
	*bla*_OXA−603_	Acquired AMR gene	4 (1.5)
	*bla*_OXA−460_	Acquired AMR gene	3 (1.1)
	*bla*_OXA−489_	Acquired AMR gene	2 (0.8)
	*bla*_OXA−591_	Acquired AMR gene	2 (0.8)
	*bla*_OXA−447_	Acquired AMR gene	1 (0.4)
	*bla*_OXA−449_	Acquired AMR gene	1 (0.4)
	*bla*_OXA−577_	Acquired AMR gene	1 (0.4)
	*bla*_OXA−631_	Acquired AMR gene	1 (0.4)
Aminoglycosides	*aadE (ant(6)-Ia)^b^*	Acquired AMR gene	49 (18.6)
	*aph(3′)-IIIa^c^*	Acquired AMR gene	2 (0.8)
	*aad9^d^*	Acquired AMR gene	1 (0.4)
	*sat4^e^*	Acquired AMR gene	2 (0.8)
Macrolides	50S *rRNA* L22-A103V	Point mutation	24 (9.1)
	23S *rRNA* A2075G	Point mutation	2 (0.8)
Arsenic	*acr3*	Acquired metal resistance	82 (31.2)
	*arsP*	Acquired metal resistance	182 (69.2)
Multidrug efflux transporter	*cmeABC*		263 (100%)

a *In 16 isolates a bla_OXA_ gene was detected, but coverage or identity was <100% (coverage range 93.39–100%, identity range 94.76–100%)*.

b *Associated with streptomycin resistance*.

c *Associated with kanamycin and amikacin resistance*.

d *Associated with spectinomycin resistance*.

e *Associated with streptothricin resistance*.

A variety of 12 known β lactam resistance genes (*bla*_OXA_ variants) were detected by AMRFinderPlus in 241 strains (92%), the most prevalent being *bla*_OXA−193_, *bla*_OXA−461_, and *bla*_OXA−580_ (56, 16, and 7%, respectively) (Table 1). The G → T transversion in the −10 promoter region of *bla*_OXA−193_, which confers high-level β lactam resistance in *C. jejuni* (Zeng et al., 2014; Lopes et al., 2019), was found in 53 (20%) isolates.

The *gyrA* T86I point mutation predicting reduced susceptibility to fluoroquinolones (Shen et al., 2017) was highly prevalent and was detected in 76% of the isolates by AMRFinderPlus (Table 1). In addition, 19 mutations in GyrA were also detected using the BioNumerics software. The number of mutations in a single isolate ranged from 1 to 6.

The 23S *rRNA* A2075G point mutation conferring high-level macrolide resistance (Corcoran et al., 2006) was found in two isolates only (0.8%). The 50S *rRNA* L22-A103V point mutation in the L22 protein was detected in 9.1% of the isolates. This mutation has been reported in both susceptible and resistant *Campylobacter* strains (Rozynek et al., 2013; Lehtopolku et al., 2020) (Table 1).

Additionally, the operon of the *cmeABC* multidrug efflux transporter, consisting of *cmeA, cmeB*, and *cmeC* genes, was present in all 263 isolates (Table 1). This transporter is the predominant efflux system in *Campylobacter* and contributes to resistance to structurally diverse agents. Inactivation of *cmeABC* results in susceptibility to tetracyclines and fluoroquinolones (Shen et al., 2017).

A high prevalence of the arsenic resistance genes *acr3* and *arsP* was observed. The *arsP* organoarsenical efflux permease (Shen et al., 2017) was found in 182 strains (69%). The presence of *arsP* is associated with elevated MICs to the arsenic compound roxarsone. The *acr3* gene, encoding an arsenite efflux transporter (Shen et al., 2017), was found in 82 strains (31%). Both genes coexisted in 80 isolates.

### Phenotypic Determination of AMR

The phenotypic AMR to six antimicrobial agents, determined by broth microdilution, is presented in Table 2. Among the 219 tested isolates, phenotypic antimicrobial resistance rates were as follows: tetracycline, 84.5%; ciprofloxacin, 77.6%; nalidixic acid, 77.2%; streptomycin, 17.8%; and erythromycin, 0.9%. Resistance to gentamicin was not detected. Only 16 (7.3%) isolates were susceptible to all six tested antibiotic agents, while the rest were resistant to between one and five agents (Table 3). More than half of the isolates were resistant to both quinolones and tetracycline (51.6%), and 16.9% were multidrug resistant.

**Table 2:** Minimum inhibitory concentration (MIC) distribution among 219 *C. jejuni* isolates^a^.

**Antibiotic agent**	**Resistance (%)**	**MIC**, **μg/ml**														
		**Breakpoint**	**Range**	**<0.12**	**0.12**	**0.25**	**0.5**	**1**	**2**	**4**	**8**	**16**	**32**	**64**	**128**	**>128**
Ciprofloxacin	77.6	>0.5	0.12–16	46		1	2	1	1	15	48	83	22			
Nalidixic acid	77.2	>16	1–64						2	32	14	2	5	29	135	
Tetracycline	84.5	>2	0.5–64			33			1	8	4	16	15	58	84	
Erythromycin	0.9	>4	1–128					217							1	1
Streptomycin	17.8	>4	0.25–16			1	2	67	11	1	1	37				
Gentamicin	0	>2	0.12–16	3		74	14	1	1							

a *Red font denotes resistance range, gray shadow denotes minimum and maximum ranges*.

**Table 3:** Phenotypic resistance profiles determined by broth microdilution among 219 *C. jejuni* isolates.

**Resistance profile^a^**	**No. isolates (%)**
Susceptible	16 (7.3)
Nal, Cip, Tet	113 (51.6)
Str, Nal, Cip, Tet	35 (16.0)
Tet	31 (14.2)
Nal, Cip	18 (8.2)
Ery, Str, Nal, Cip, Tet	2 (0.9)
Cip, Tet	2 (0.9)
Str, Tet	1 (0.5)
Nal, Tet	1 (0.5)
Multidrug resistance^b^	37 (16.9)

a *Tested for six antimicrobial agents: nalidixic acid (Nal), ciprofloxacin (Cip), tetracycline (Tet), streptomycin (Str), erythromycin (Ery), and gentamicin (Gen)*.

b *Resistance to three or more classes of antibiotics*.

### Comparison Between Genomic Prediction and Phenotypic Determination of AMR

Comparison between WGS-based genotypic predictions of AMR and microdilution-based phenotypic AMR was based on 1,314 susceptibility tests for 219 isolates also screened for the presence of AMR genetic determinants. The overall correlation rate was 98.8%, with a sensitivity of 98.0%, specificity of 99.3%, PPV of 99.1%, and NPV of 98.5% (Table 4). There were 16 (1.2%) discrepancies, mostly associated with an absence of resistance determinants among phenotypically resistant isolates. The highest number of discrepancies was noted for tetracycline, with six (2.7%) of the phenotypically resistant isolates reported as negative for the *tet(O)* gene. Further analysis of these discrepancies by BLAST indicated that in four strains, *tet(O)* is present, but it is fragmented among small contigs and in different strands, and therefore is not identified by AMRFinderPlus. Therefore, we attribute 4 discrepancies to assembly fragmentation, while 2 discrepancies could not be accounted for. A combination of the assembly-based approach with read-based mapping may prove useful in minimizing discrepancies due to gene fragmentation.

**Table 4:** Comparison between microdilution-based phenotypic resistance and genotype-predicted resistance according to AMR genetic determinant for 219 *C. jejuni* isolates.

**Antibiotic agent**	**Phenotypic profile**	**No. isolates (%)**	**AMR determinant detected**	**AMR gene present/absent**	**Correlation rate^a^ (%)**	**Sen.^b^ (%)**	**Spec.^c^ (%)**	**PPV^d^ (%)**	**NPV^e^ (%)**
Ciprofloxacin	Resistant	170 (77.6)	*gyrA T86I*	168/2	98.2	98.8	95.9	98.8	95.9
	Susceptible	49 (22.4)		2/47					
Nalidixic acid	Resistant	169 (77.2)	*gyrA T86I*	168/1	98.6	99.4	96.0	98.8	98.0
	Susceptible	50 (22.8)		2/48					
Streptomycin	Resistant	38 (17.4)	*aadE*	36/2	99.1	94.7	100.0	100.0	98.9
	Susceptible	181 (82.6)		0/181					
Gentamicin	Resistant	0	*aph(3′)-IIIa*	0/0	100.0	100.0	100.0	100.0	100.0
	Susceptible	219 (100)		0/219					
Erythromycin	Resistant	2 (0.9)	23S *rRNA* A2075G	2/0	100.0	100.0	100.0	100.0	100.0
	Susceptible	217 (99.1)		0/217					
Tetracycline	Resistant	185 (84.5)	*tet(O)*	179/6	96.8	96.8	97.1	99.4	84.6
	Susceptible	34 (15.5)		1/33					
Total	Resistant	564 (42.9)		553/11	98.8	98.0	99.3	99.1	98.5
	Susceptible	750 (57.1)		5/745					

*AMR, Antimicrobial resistance; Sen., Sensitivity; Spec., Specificity; PPV, Positive predictive value; NPV, Negative predictive value*.

a *Calculated as the sum of true positive and true negative divided by all tested isolates*.

b *Calculated by dividing true positive by the sum of true positive and false positive*.

c *Calculated by dividing true negative by the sum of true negative and false negative*.

d *Calculated by dividing true positive by the sum of true positive and false negative*.

e *Calculated by dividing true negative by the sum of true negative and false positive*.

**Figure 2:**
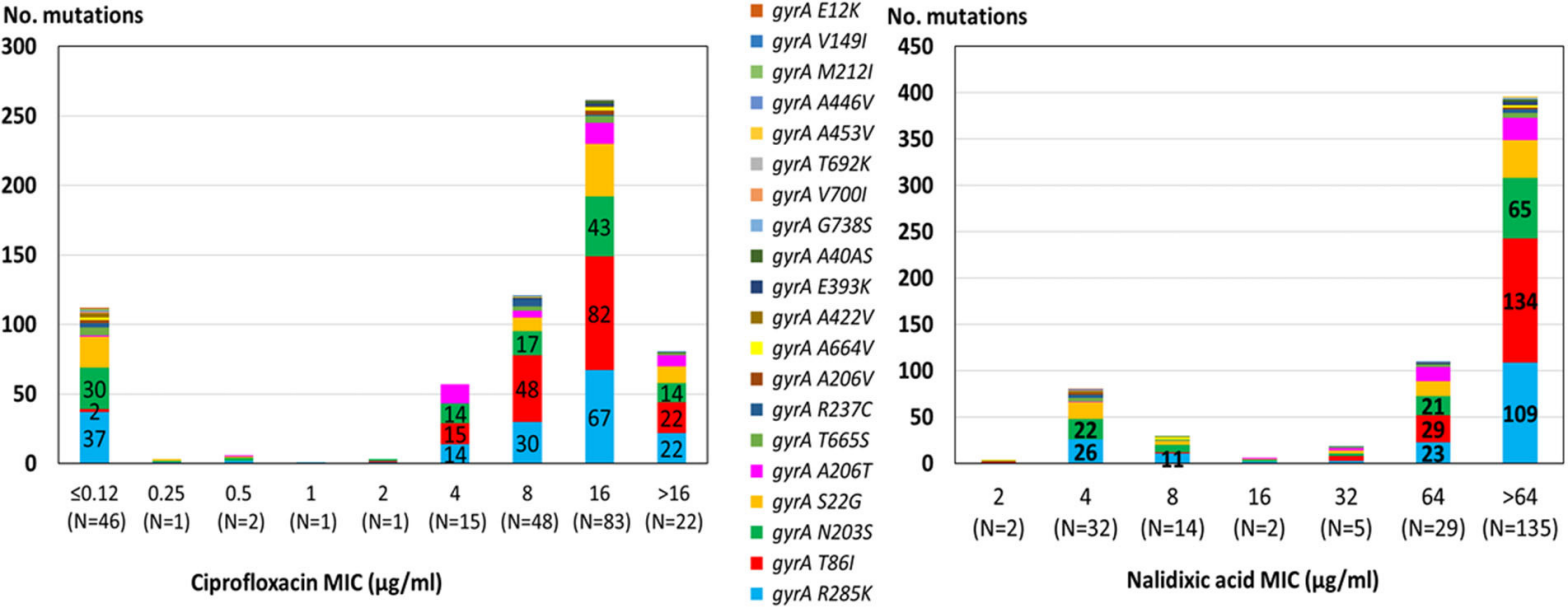
Correlation between quinolone MIC and the presence of point mutations in the GyrA protein among 219 *C. jejuni* isolates. The different mutations are denoted by color. Resistance breakpoints: ciprofloxacin (>0.5 μg/ml), nalidixic acid (>16 μg/ml).

The highest correlation rate was found for erythromycin and gentamicin. The two isolates with the 23S *rRNA* A2075G point mutation were phenotypically highly resistant to erythromycin (MIC ≥ 128 μg/ML). All isolates were susceptible to gentamicin and no resistance determinant to gentamicin was detected. The correlation rate of ciprofloxacin and nalidixic acid genotypic prediction was also high, reaching 98.2 and 98.6%, respectively. Only the *gyrA* T86I point mutation showed this high correlation rate, while all the other *gyrA* point mutations were present in both resistant and susceptible isolates (Figure 2). Notably, the *gyrA* T86I point mutation was present across the whole range of resistance to ciprofloxacin and nalidixic acid, and the number of the additional point mutations per isolate was not correlated with the level of resistance. Similarly, the range of resistance to tetracycline and streptomycin could not be explained by the single resistance determinant detected for each agent.

### Distribution of AMR Determinants Across Genetic Clusters

**Figure 3:**
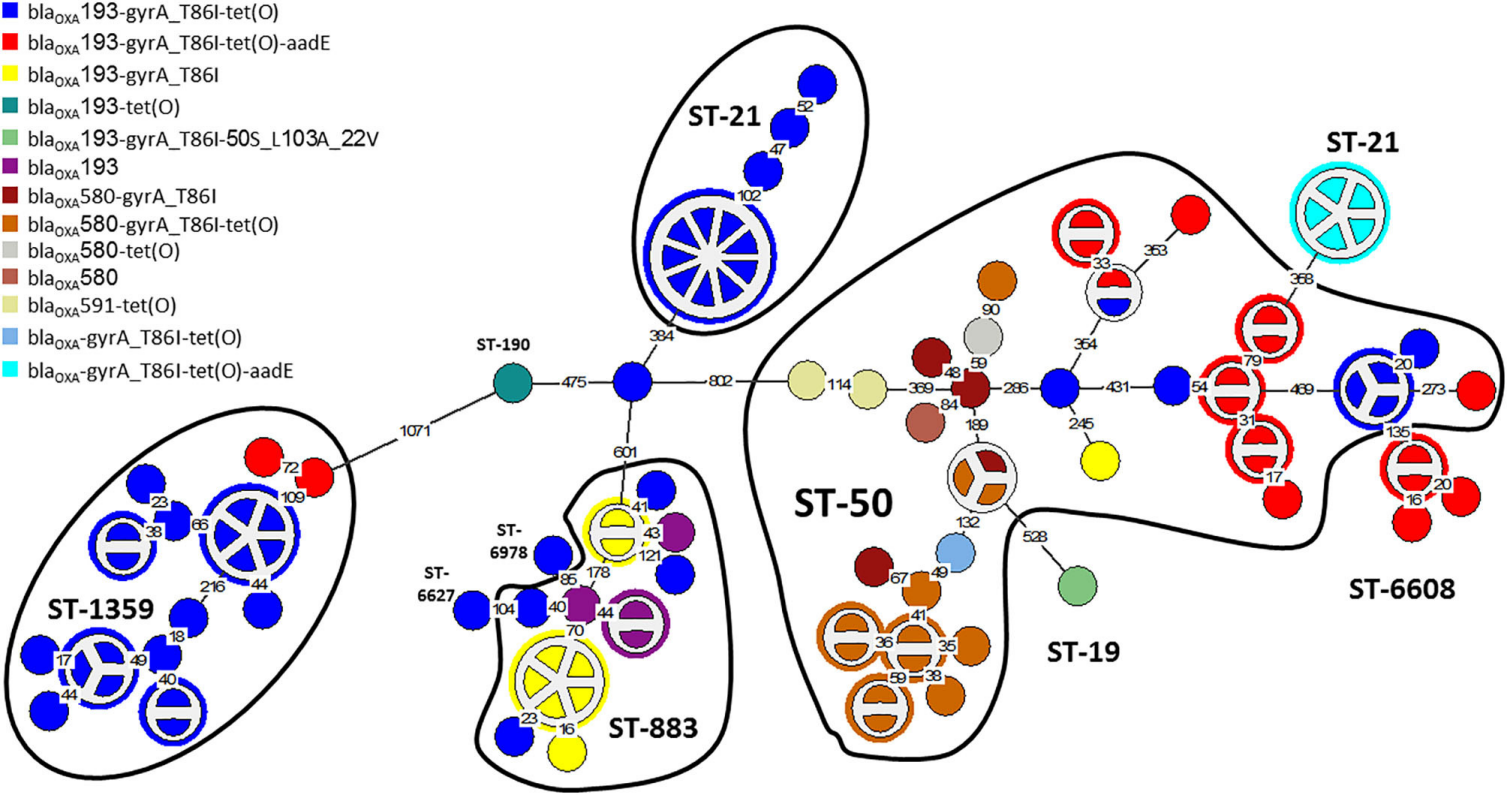
AMR genetic determinants among a wgMLST phylogeny of Clonal Complex 21. A wgMLST-based minimum spanning tree of 104 CC-21 isolates is shown. Each node represents a strain. Partitioned nodes represent closely clustered isolates (≤ 15 allelic distance threshold). Sequence types are denoted in black. The number of allelic differences is shown on the branches connecting the nodes. The AMR gene profile detected by AMRFinderPlus is denoted by color.

The distribution of resistance determinants among lineages and within genetic clusters was further investigated. wgMLST-based phylogeny indicated a high level of clonality and clustering among the studied population. Among 263 isolates, 132 isolates (50.2%) formed 38 genetic clusters (single linkage distance ≤ 15 alleles). Cluster sizes ranged between 2 and 13 strains (Figure 1). We investigated the distribution of AMR genes within clades and clusters. Figure 3 depicts the presence of AMR determinants among wgMLST phylogeny of CC-21, the largest clonal complex. A variety of AMR profiles were detected among CC-21 sequences. The predominant STs (ST-21, ST-50, ST-1359, ST-883) were each associated with more than one AMR profile (2–9 profiles).

**Figure 4:**
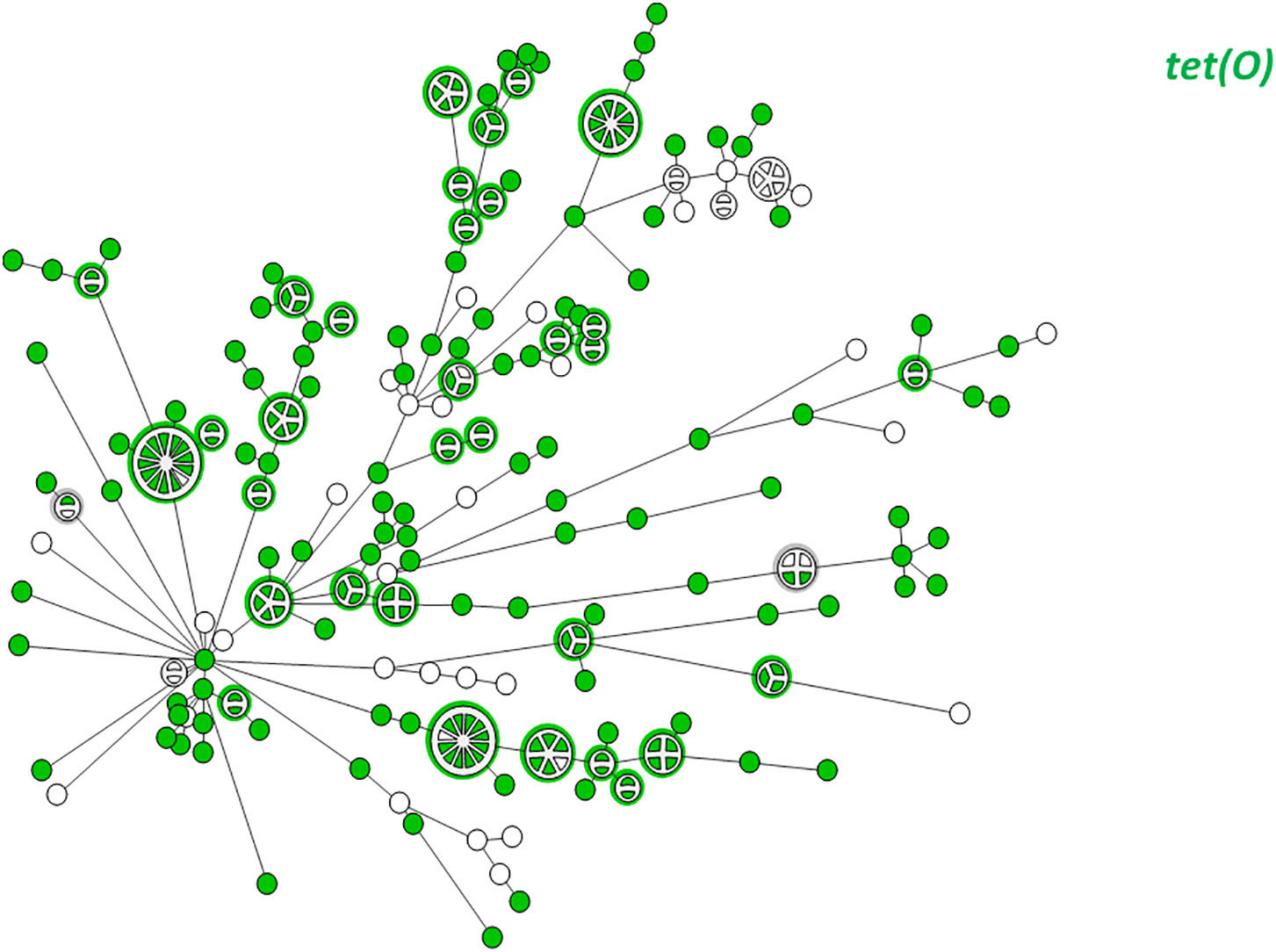
wgMLST-based phylogeny of 263 *C. jejuni* isolates. The distribution of the *tet(O)* gene associated with tetracycline resistance is shown in color.

**Figure 5:**
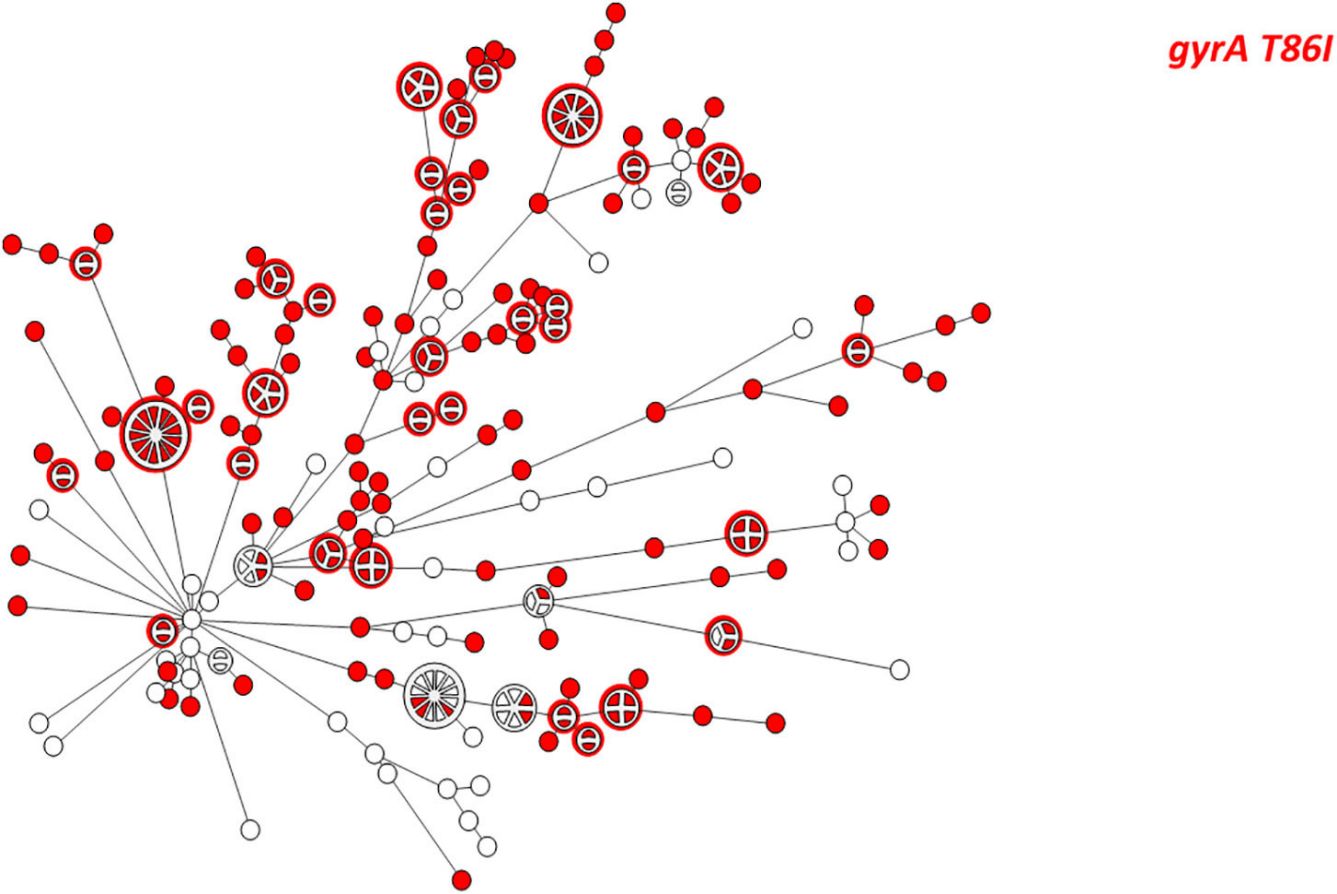
wgMLST-based phylogeny of 263 *C. jejuni* isolates. The distribution of the *gyrA* T86I point mutation associated with quinolone resistance is shown in color.

**Figure 6:**
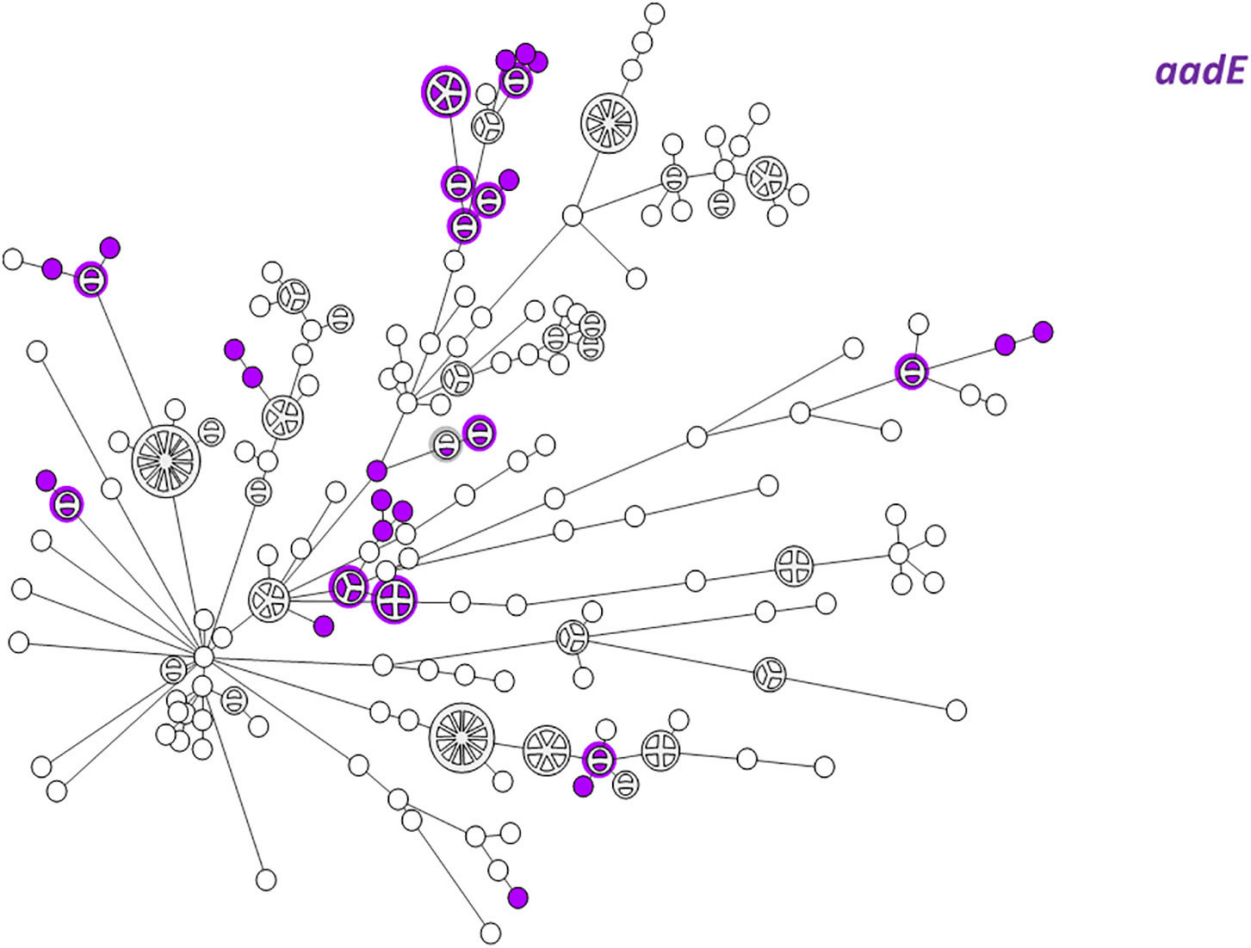
wgMLST-based phylogeny of 263 *C. jejuni* isolates. The distribution of the *aadE (ant(6)-Ia)* gene associated with streptomycin resistance is shown in color.

**Figure 7:**
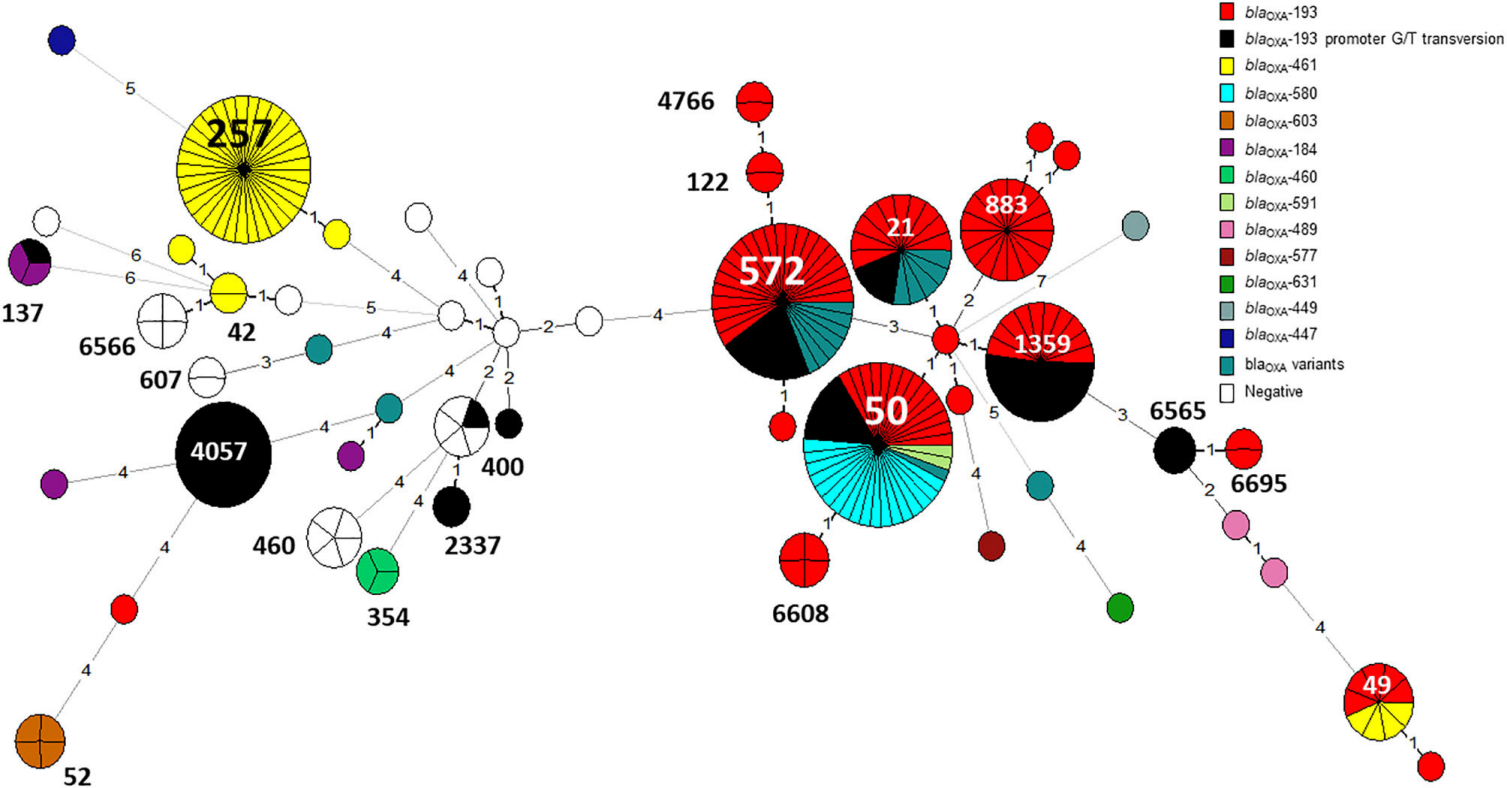
The distribution of *bla*_OXA_ gene variants across an MLST-based phylogeny of 263 *C. jejuni* isolates. Each node in the minimum spanning tree represents a sequence type (ST), and the number of allelic differences is denoted on the branches connecting the nodes. The presence of β-lactamase (*bla*_OXA_) gene variants is denoted by color. The G → T promoter transversion associated with high-level ampicillin resistance in *bla*_OXA−193_ is shown in black.

WGS-based clustering was predictive of AMR gene content. Closely related isolates that were part of a genetic cluster also shared a homogenous AMR determinant profile. This was observed in 18 of 20 (90.0%) clusters in CC-21. These results may reflect clonal expansion of resistant isolates. Phylogenetic analysis reflected varied patterns of antimicrobial gene distribution across the studied isolates. The prevalent *tet(O)* gene and the *gyrA* T86I point mutation were evenly distributed across the wgMLST clusters (Figures 4, 5). The *gyrA* T86I mutation was less prevalent in some clusters within CC-257 and CC-206. Strong lineage association was noted for the *aadE* (Figure 6) gene and the various *bla*_OXA_ genes (Figure 7). For example, the *bla*_OXA−580_ gene was uniquely found in ST-50 and the *bla*_OXA−461_ was predominantly found in ST-257.

The G → T transversion in *bla*_OXA−193_ was detected mainly in ST-4057 and ST-1359 strains (Figure 7). The aminoglycoside resistance gene cluster *aadE (ant(6)-Ia- aph(3')-IIIa-sat4* was detected in two isolates of ST-2337 from bacteremic patients.

### Temporal Trends in the Occurrence of AMR Determinants

**Figure 8:**
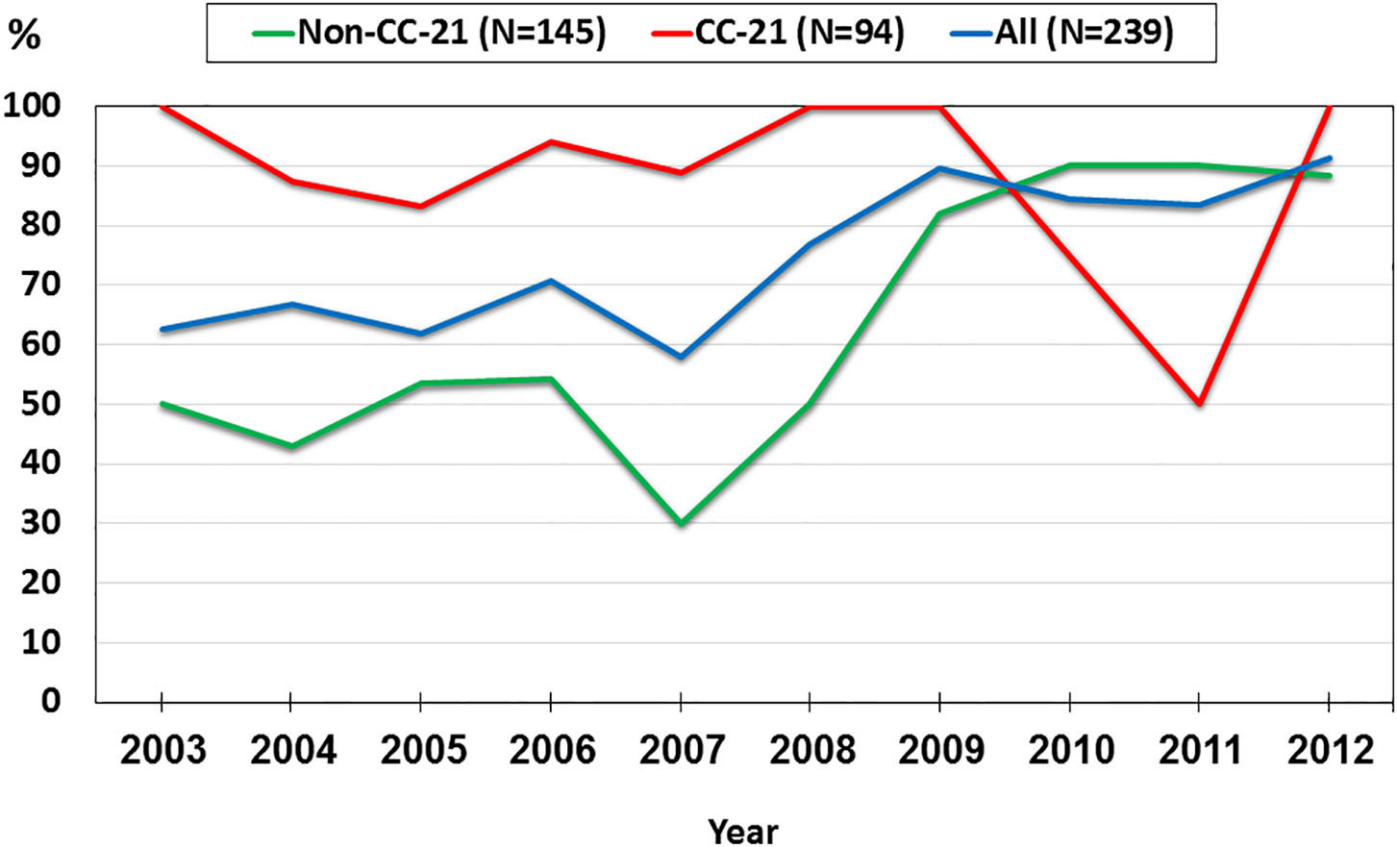
Trends in the prevalence of the *gyrA* T86I point mutation among 239 human *C. jejuni* isolates collected over a decade.

**Figure 9:**
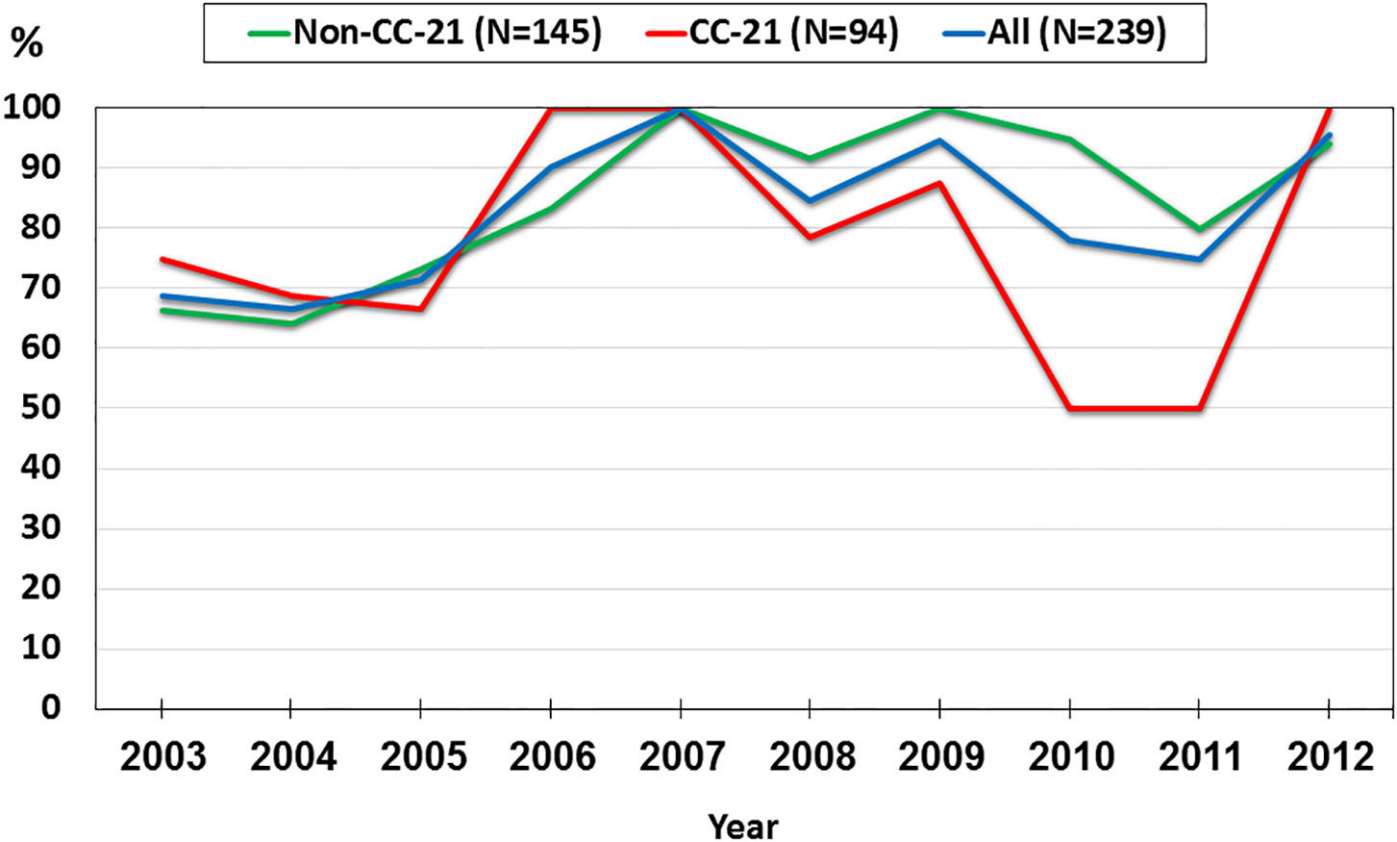
Trends in the prevalence of the *tet(O)* gene among 239 human *C. jejuni* isolates collected over a decade.

We investigated trends in the presence of AMR determinants among 239 human *C. jejuni* studied isolates over a 10-year period (2003–2012). The prevalence of *gyrA* T86I point mutation increased from 62.5% in 2003 to 91.3% in 2012 (a 1.5-fold increase). This trend differed among the various lineages. Among CC-21 isolates, the prominent clonal complex in Israel, *gyrA* T86I detection rate was high and stable along the study period, and as high as 100% in several years, while in non-CC-21 lineages detection increased 1.7-fold (from 50.0% in 2003 to 88.2% in 2012) (Figure 8). The detection of *tet(O)* gene increased 1.4-fold during the study period from 68.8 to 95.7%; no substantial difference was noted between CC-21 and the other CCs (Figure 9). These trends did not reach statistical significance and warrant further investigation on a larger scale.

## Discussion

This study explored resistance trends among Israeli *C. jejuni* over more than a decade, the genetic basis for resistance phenotypes, and the association of phenotypic and genotypic resistance with phylogeny. The study indicates an alarmingly high rate of quinolone and tetracycline resistance among the Israeli *C. jejuni* strains emerging over the last decade, and a low resistance rate to macrolides and aminoglycosides. These rates are higher than those reported by NARMS for the United States and the average rates reported by the EU, but comparable with the rates in several EU countries such as Spain, Portugal, Lithuania, or Cyprus (CDC, 2018; European Food Safety Authority and European Centre for Disease Prevention and Control, 2019). Current resistance rates at certain localities in Israel may be even higher (Azrad et al., 2018).

We found a high correlation rate between the presence of AMR determinants detected by the NCBI AMRFinderPlus tool and the phenotypic resistance. The overall correlation rate was 98.8%, with a sensitivity of 98.0%, specificity of 99.3%, PPV of 99.1%, and NPV of 98.5%. Comparable results were reported by Feldgarden et al. for *C. jejuni*, using the NCBI AMRFinder tool, e.g., 98.9% correlation rate, with a PPV of 97.1% and NPV of 99.2% (Feldgarden et al., 2019). A high correlation rate of 97.5% was also found in a most recent study from England and Wales using another AMR extraction tool, GeneFinder (Painset et al., 2020).

The overall discordance rates between genetic prediction and phenotypic AMR for *C. jejuni* was low in our study (1.2%), as well as in the above two studies. The highest discordance rate (7 out of 219, 3.2%) in our study was noted for tetracycline, and particularly for resistant isolates lacking the *tet(O)* gene (6/219, 2.7%). No discordance was noted for gentamicin and erythromycin. The discordance rate for the other antibiotic agents (ciprofloxacin, tetracycline, streptomycin) was <2% (Table 4). Feldgarden et al. and Painset et al. also found 100% consistency for erythromycin based on the presence/absence of mutations in the 23S *rRNA*, and a low rate of genotype-phenotype discordances for fluoroquinolones, macrolides, and aminoglycosides (Feldgarden et al., 2019; Painset et al., 2020). An exception was resistance to streptomycin in the study by Painset et al., as only 2 of 5 isolates carrying the streptomycin resistant gene (*aadE*) were phenotypically resistant to the drug. In our study, 36 isolates resistant to streptomycin carried the *aadE* resistance gene, but it was lacking in two other resistant phenotypes.

There are multiple explanations for possible WGS-based genotypic-phenotypic discrepancies, related to the quality of assembly, bioinformatics tools, the completeness, and curation of WGS-based AMR database, and factors related to the studied isolate, namely gene content, transcriptome and proteome, and plasmid stability during microbiological procedures (Zankari et al., 2017; Chen et al., 2019; Doyle et al., 2020). Low quality of assemblies may lead to defective genes, resulting in incorrect extraction from the AMR databases (Feldgarden et al., 2019; Doyle et al., 2020). Long-read sequencing technology can improve complete genome assembly accuracy with the addition of plasmid sequencing (Chen et al., 2019). Indeed, the current short-read assembly technologies used for AMR detection may miss genetic determinants carried on plasmids, mainly due to repetition or redundancy (Collineau et al., 2019).

The performance of web-based AMR search tools is also important for the prediction of phenotypes. For example, a comparison between AMRFinder and ResFinder (Feldgarden et al., 2019) showed miscalling of aminoglycosides resistance genes due to lack of correspondence between the closest nucleotide hit and actual observed sequence. In other instances ResFinder overspecified, while AMRfinder underspecified the gene symbols, resulting in decreased genotype-phenotype correlation (Feldgarden et al., 2019). Another study took it one step further and compared the results of AMR phenotype prediction of the same bacterial sequences among nine different laboratories (Doyle et al., 2020). It showed a high degree of discordance of genotype-phenotype prediction and substantial variability between the participating laboratories, which stemmed from multiple factors, including the quality of the sequence data, the choice of bioinformatic pipeline, and the interpretation of the results. This study highlighted the understanding that genotypic AMR determination requires standardization of sequencing methods, the AMR databases, and the bioinformatics tools.

At the pathogen level many factors may influence lack of AMR genotype-phenotype correlation, such as the number of alleles, overexpression and under expression of genes (e.g., efflux pumps), mutations in regulatory proteins or their binding sites, protein abundance levels, and contributing factors that are not covered by the searched database (e.g., porin mutations). Finally, novel resistant genes and mutations may be missed by the current databases and search tools (Zankari et al., 2017; Chen et al., 2019; Feldgarden et al., 2019). This may also explain the lack of correlation between the presence of a single resistance determinant and a range of MICs for a specific antibiotic.

The trend of increasing prevalence of resistance determinants to quinolones and tetracycline along the study years is of concern. Similar trends have been reported globally and linked to extensive use of clinically important antibiotics in veterinary medicine (Takahashi et al., 2005; Tang et al., 2017). Monitoring these trends is important for decisions on the choice of appropriate treatment of clinical infections as well as intervention policies. These results also call for a better control of antibiotic-resistant *C. jejuni* in Israel.

The wgMLST phylogenetic analysis indicates that the *C. jejuni* population in Israel consists of diverse lineages and genotypes. Notably, half of the strains were part of genetic clusters (≤ 15 allelic distance), indicative of the dominance of several different clones within the population. Clonality may reflect common source or diffuse outbreaks as well as selective expansion of successful clones over time and space. Our analysis shows a link between phylogeny and AMR profile. Among CC-21, closely clustered isolates contained uniform AMR arsenals (18 of 20 clusters). Therefore, the AMR profile could be correctly predicted based on wgMLST clustering in 90% of clusters.

Our study is limited by the small sample size of isolates selected to represent a decade of surveillance. More recent isolates may exhibit a higher rate of resistance, but this is not expected to influence the predictive value of genotypic-phenotypic correlations.

In conclusion, our study illuminates the genetic mechanisms underlying AMR in *C. jejuni in* Israel. We report a high rate of tetracycline and quinolone resistance among the Israeli *C. jejuni* strains and a low resistance rate to macrolides and aminoglycosides. Some AMR determinants were associated with distinct genotypes and clones. Our study revealed a high correlation rate between the presence of AMR determinants found by the NCBI AMRFinderPlus tool and phenotypic resistance. However, some resistance phenotypes could not be predicted by the presence of AMR determinants and particularly not the level of resistance expressed as MIC. Further work to improve phenotypic prediction of AMR is central to the envisaged transition toward WGS analysis as the primary approach for characterizing foodborne isolates.

## Data Availability Statement

The datasets generated for this study can be found in the Campylobacter PubMLST database, IDs 76153-76415.

## Ethics Statement

The studies involving human participants were reviewed and approved by the local Ethical Committee at Shamir (Assaf Harofeh) Medical Center, Zerifin (#160/11), Israel and the local Ethical Committee of Tel Aviv University, Tel Aviv, Israel. Written informed consent from the participants' legal guardian/next of kin was not required to participate in this study in accordance with the national legislation and the institutional requirements.

## Author Contributions

AR, LV, JM-G, VA, and MW conceived the study. AR, LV, and NF performed the laboratory analysis. AR performed the bioinformatic analysis for antibiotic resistance determinants. AR, KV, and MW performed the data analyses. AR and MW drafted the manuscript with help from the other authors. JM-G critically revised the data and the manuscript. All authors read and approved of the submitted version.

## Conflict of Interest

KV was employed by Applied Maths NV. The remaining authors declare that the research was conducted in the absence of any commercial or financial relationships that could be construed as a potential conflict of interest.

## Supplementary Material

Supplementary Table 1 List of isolates included in the study, metadata and sequence parameters.

Supplementary Table 2 Genotypic antimicrobial resistance determinants and phenotypic broth microdilution results.
